# The Impact of Myopia on Regional Visual Field Loss and Progression in Glaucoma

**DOI:** 10.1167/tvst.14.9.34

**Published:** 2025-09-24

**Authors:** Anagha Lokhande, Luo Song, Yueyin Pang, Yan Luo, Louis R. Pasquale, Sarah R. Wellik, Carlos Gustavo De Moraes, Jonathan S. Myers, Mohammad Eslami, Tobias Elze, Lucy Q. Shen, Nazlee Zebardast, David S. Friedman, Michael V. Boland, Mengyu Wang

**Affiliations:** 1 Harvard Ophthalmology AI Lab, Schepens Eye Research Institute of Mass Eye and Ear, Harvard Medical School, Boston, MA, USA; 2 The Warren Alpert Medical School of Brown University, Providence, RI, USA; 3 Eye and Vision Research Institute, Icahn School of Medicine at Mount Sinai, New York, NY, USA; 4 Bascom Palmer Eye Institute, University of Miami School of Medicine, Miami, FL, USA; 5 Edward S. Harkness Eye Institute, Columbia University Medical Center, New York, NY, USA; 6 Wills Eye Hospital, Thomas Jefferson University, Philadelphia, PA, USA; 7 Mass Eye and Ear, Harvard Medical School, Boston, MA, USA

**Keywords:** glaucoma, myopia, functional vision loss, visual fields (VFs)

## Abstract

**Purpose:**

The purpose of this study was to investigate the impact of myopia on regional visual field (VF) loss and progression in glaucoma.

**Methods:**

We included 112,633 24-2 VFs; longitudinal analyses comprised patients with at least 5 reliable VFs over 4 years. The degree of myopia was measured by spherical equivalent (SE) extracted from VF testing. Linear and Cox regressions determined the impact of myopia on regional VF loss and progression, respectively. We calculated three VF progression outcomes: (1) mean deviation (MD) progression: MD slope <0; (2) total deviation (TD) pointwise progression: at least 3 TD locations with TD slope ≤−1 decibels (dB)/year; (3) MD fast progression: MD slope ≤−1 dB/year (*P* value < 0.05). Longitudinal analyses were conducted for all subjects and with exclusion of patients with high myopia (SE ≤−6.00 diopters [D]).

**Results:**

More negative SE values were associated with worse TD values in the paracentral VF region (up to −0.14 dB/D). A more negative SE is associated MD (odds ratio [OR] = 0.95), TD pointwise (OR = 0.96), and MD fast progression (OR = 0.94; *P* < 0.001). Results were comparable when excluding patients with high myopia (*P* < 0.001): MD (OR = 0.95), VFI (OR = 0.95), and MD fast progression (OR = 0.94).

**Conclusions:**

Lower SE values are associated with worse paracentral VF loss. Worse myopia is associated with functional progression, even when excluding patients with high myopia.

**Translational Relevance:**

We provide evidence for the relationship between SE and VF progression and inform clinical practice by highlighting even mild myopia as a highly prevalent possible risk factor for glaucoma progression.

## Introduction

Glaucoma, a progressive optic neuropathy, is one of the leading causes of irreversible blindness in adults worldwide.^1^ Myopia is the leading cause of distance refractive error globally, and its prevalence is increasing, particularly among young people.^2^^,^^3^ The association between myopia and the prevalence of glaucoma is well-documented.^4^^,^^5^ Myopic eyes are structurally different from emmetropic or hyperopic eyes with increased axial length, optic disc tilt, and torsion.^6^^–^^10^ Prior work has explored the relationship between myopia and regional visual field (VF) loss in glaucomatous eyes, finding that myopic eyes have their major retinal nerve fiber layer (RNFL) bundles closer to the macula than hyperopic eyes. Consequently, glaucomatous optic nerve damage in myopic eyes has been shown to produce greater VF loss in the paracentral region (i.e. closer to central vision) whereas hyperopic eyes tend to demonstrate greater VF depression in the temporal region.^11^

The association between myopia and VF progression in glaucoma remains an open question, with some prior work reporting no association.^12^^,^^13^ One study explored the relationship between myopia and VF progression among eyes with any degree of myopia and suggested an increasing prevalence of VF progression in eyes with increasingly severe myopia, across all levels of myopia.^14^ Another study suggested that high myopia, defined in this study as more than four diopters (D), but not low to moderate myopia, is a risk factor for VF progression in primary open-angle glaucoma.^15^ However, a study investigating normal tension glaucoma reports no association between high myopia and VF progression.^16^ Other findings in cohorts of patients with primary open-angle glaucoma report no association between any level of myopia and VF progression.^12^ Conversely, other studies in normal tension glaucoma reported that myopia may be a protective factor against VF progression.^17^^,^^18^ Furthermore, several prior studies have attempted to study risk factors for fast progression and found that myopia is associated with fast progression.^19^^–^^21^ The sample sizes used in these studies (on the order of a few hundred patients at most) might explain these conflicting findings, as suggested by Lee et al.^12^ Particularly given its increasing prevalence, determining the extent to which myopia is associated with regional VF deficits or increased probability of VF progression in glaucoma may be of increasing clinical relevance to the management of affected patients.

Our large multicenter study aims to build upon previous studies further exploring if and how myopia is associated with regional VF field loss, using cross-sectional analyses, and VF progression, using longitudinal analyses, in glaucoma suspect eyes. Our study may aid clinicians in better identifying VF loss patterns and in better prognosticating VF progression for patients with glaucoma.

## Methods

This retrospective cross-sectional and longitudinal study was conducted using VFs from the Glaucoma Research Network performed between 1999 and 2014. The Glaucoma Research Network consists of the following institutions: Massachusetts Eye and Ear, Wilmer Eye Institute, New York Eye and Ear Infirmary of Mount Sinai, Bascom Palmer Eye Institute, and Wills Eye Hospital. The institutional review boards of each participating institution approved this retrospective study and granted a waiver for informed consent due to the use of deidentified patient data. This study complies with the principles of the Declaration of Helsinki and adheres to all relevant federal and state regulations, including the Health Insurance Portability and Accountability Act of 1996.

### Participants and Data

Reliable Swedish interactive thresholding algorithm standard 24-2 VFs with stimulus size III measured by the Humphrey Field Analyzer (HFA; Carl Zeiss Meditec, Dublin, CA, USA) were selected for our data analysis. Inclusion criteria were criteria for reliable VFs: fixation loss ≤33%, false-negative rates ≤20%, and false-positive rates ≤20%.^7^^,^^21^^,^^22^ The total deviation (TD) values from each of the 52 locations tested in the 24-2 pattern and mean deviation (MD) measured in decibels (dB) were used for data analyses. All VF results were analyzed in the right-eye format. Importantly, the Glaucoma Research Network does not include information about demographics, baseline intraocular pressure (IOP), or whether a patient’s eyesight is spectacle or contact lens (CL) corrected.

### Statistical Analyses

Cross-sectional analysis was conducted using multivariable linear regression models. The main independent variables were refractive error parameters (taken from VF measurements) including spherical equivalent (SE) or sphere. SE values were exact prescription values, not ones with HFA-derived age-expected presbyopia. Distance sphere and cylinder values entered by technicians during the VF tests were exported from the Zeiss FORUM database for our study. SE was calculated as the sum of the sphere power and one-half of the cylinder power.^23^ The outcomes studied were each of the individual 52 TD values of the 24-2 VF. Age-adjusted regressions were performed with and without adjustment for each patient's first recorded MD, which served as a measure of their baseline VF function and baseline glaucoma severity. We randomly selected one eye per patient if both eyes were available to avoid statistical test bias from multiple samples from the same patient. Analysis was conducted using the first recorded VF for each patient.

For longitudinal analysis of all patient data available for cross-sectional analysis, we selected patients with at least 5 reliable VFs over 4 years. When both eyes from the same patients were available, we randomly selected one eye per patient. Cox regression analyses were used for longitudinal analysis. We assessed the impact of baseline myopia on the progression status at the end time point of the entire series. Compared with using the first instance of progression status over the follow-up course, using the progression status at the end time point of the entire series is expected to reduce false-positive events.^24^

Three VF progression outcomes were studied in separate regression analyses: MD progression (MD slope <0), TD pointwise progression (at least 3 TD locations with slope ≤−1 dB/year), and MD fast progression (MD slope ≤−1 dB/year).^25^^–^^27^ Age-adjusted Cox regression was used to predict progression outcomes from refractive error parameters (SE or sphere) with and without MD adjusted. Longitudinal analysis was repeated, excluding patients with high myopia (−6.00 ≤SE).^28^^,^^29^ Values are reported as odds ratios (ORs) with associated 95% confidence intervals (CIs). Kaplan-Meier curves were generated for each progression outcome, with patients grouped into myopes (SE ≤−0.5), hyperopes (SE ≥0.5), and emmetropes (−0.5 < SE <0.5). We also generated linear regressions to predict age-adjusted pointwise TD slopes using baseline SE values.

Statistical significance for all regressions was set at *P* < 0.05. All statistical analyses were conducted using R Statistical Software (version 3.6.3; R Core Team 2020).

## Results

**Figure 1.**
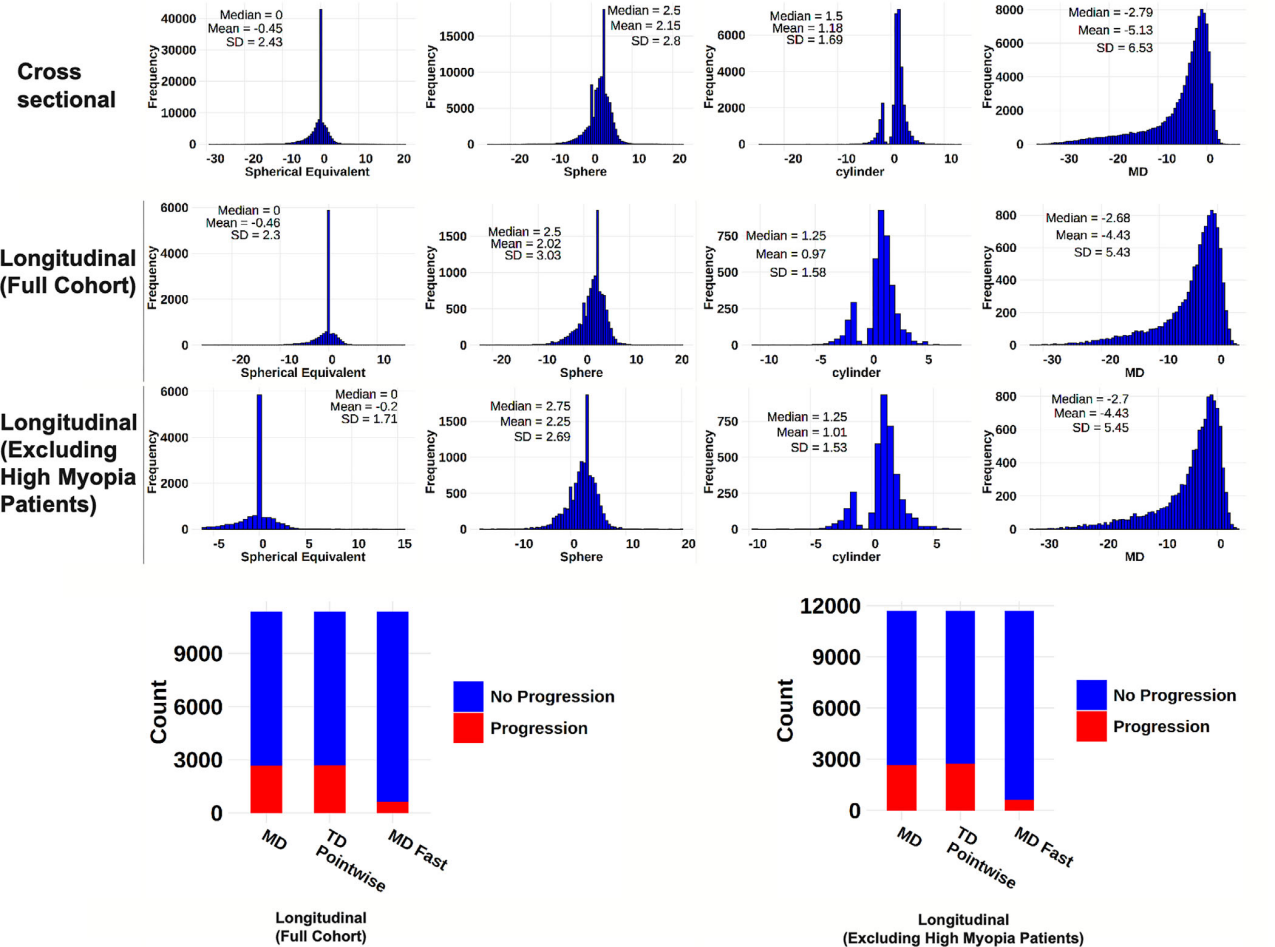
The distributions of spherical equivalent, sphere, cylinder, and MD are shown for cross-sectional analysis data. The distributions of spherical equivalent, sphere, cylinder, MD, and progression incidence rates are shown for longitudinal analysis data.

Our cross-sectional analysis sample comprises 112,633 eyes from 112,633 patients. The mean age and MD were 62.9 ± 15.9 years and −5.1 ± 6.5 decibels (dB), respectively (Fig. 1). The mean SE and sphere were −0.45 ± 2.43 D and −0.62 ± 2.49 D, respectively. For longitudinal analyses, 11,676 eyes from 11,676 patients were selected. The mean age and MD were 63.7 ± 12.7 years and −4.4 ± 5.4 dB, respectively. The mean SE and sphere were −0.55 ± 2.35 D, −0.62 ± 2.49 D, and 0.34 ± 1.01 D, respectively. When excluding high myopes (−6.00 ≤ SE), the sample size was 11,369 patients. The mean age and MD were 64.0 ± 12.6 years and −4.4 ± 5.4 dB, respectively. The mean SE and sphere were −0.20 ± 1.71 D and −0.30 ± 1.77 D, respectively.

### Cross-Sectional Analysis

**Figure 2.**
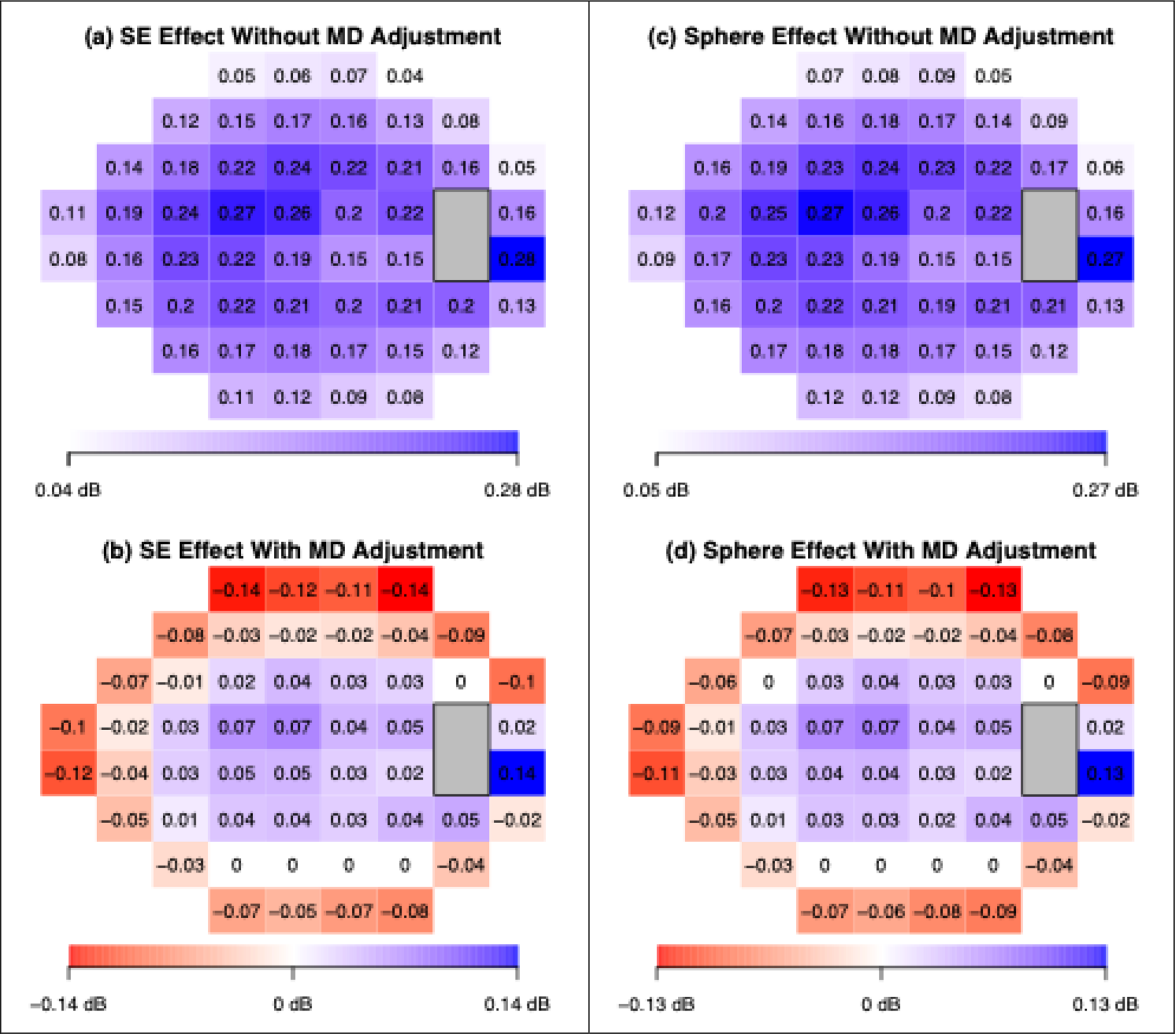
Cross-sectional analysis results. Both with and without MD adjustment, more negative SE values were associated with more negative TD values in the paracentral region (shown in *blue*). More negative SE values are associated with more positive TD values in the locations indicated in *red*. Color saturation represents the strength of association. Non-statistically significant associations were set to zero.

Cross-sectional analysis revealed that more negative SE values were associated with more negative TD values throughout the VF (Fig. 2a). These changes were seen most prominently in the paracentral region (central 16 locations around fixation). The decrease in TD values in this region ranged from 0.15 to 0.27 dB per D. This association was held with adjustment for baseline MD, with adjusted TD values ranging from 0.02 to 0.07 dB per D (Fig. 2b). Lower TD values associated with more negative SE were also seen, both with and without adjustment for MD, in the areas of the VF surrounding the physiologic scotoma.

When we correlated sphere to TD values using MD-adjusted linear regression (Fig. 2d), more negative sphere was associated with worse TD values (0.02 to 0.07 dB per D) in the paracentral region and better TD values in the peripheral region (−0.03 to −0.13 dB per D).

### Longitudinal Analysis

**Figure 3.**
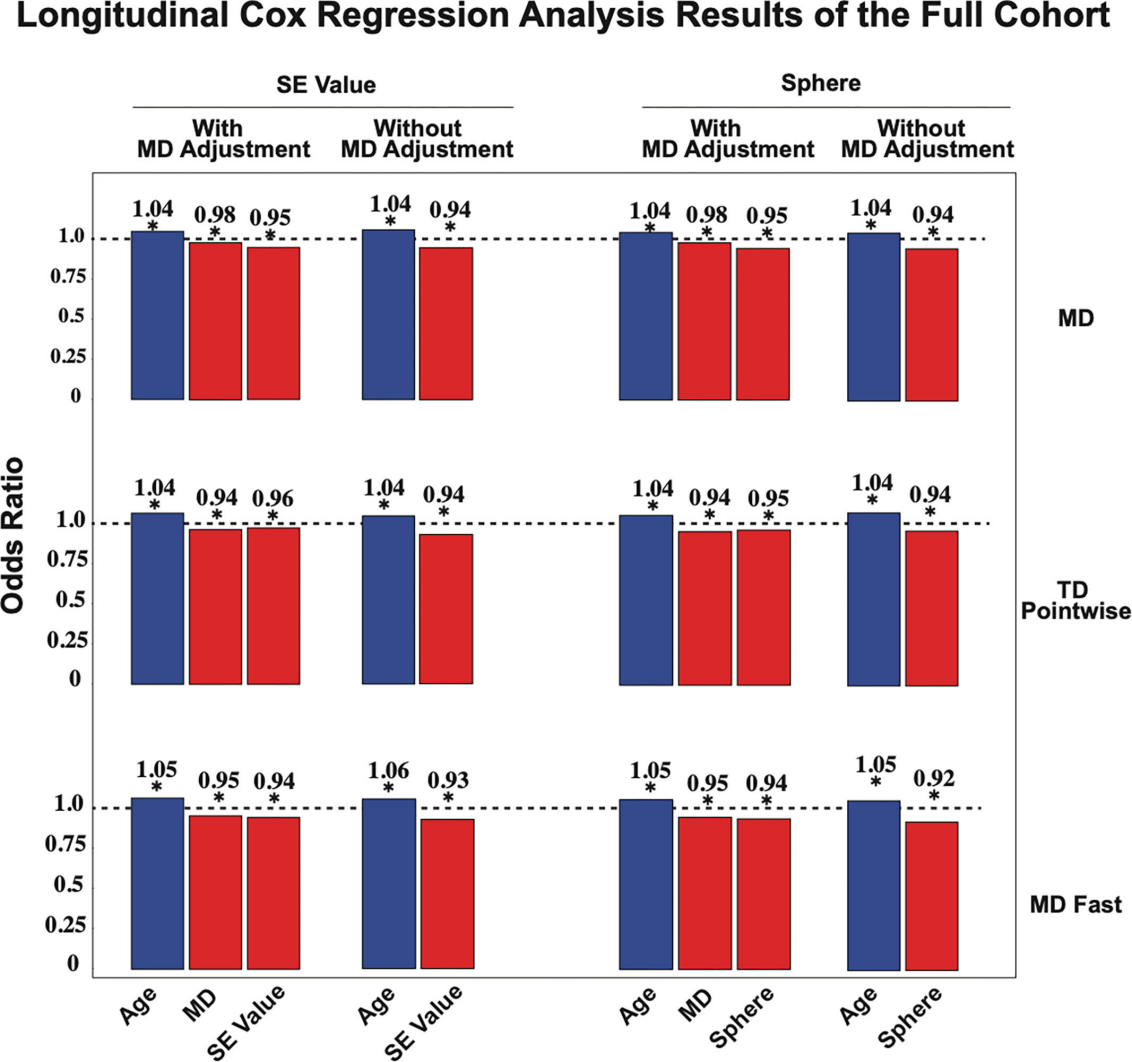
Longitudinal analysis results of the full cohort wherein and regression analysis with both adjustment and no adjustment for MD shown. Statistically significant predictors are denoted by * (*P* < 0.05).

In our longitudinal cohort, approximately 22% of patients experienced MD progression and TD pointwise progression; approximately 6% experienced MD fast progression (see Fig. 1). These results were comparable when excluding patients with high myopia (SE ≤−6.00 D). In analyses with MD adjustment among the full cohort, all three measures of VF progression had a higher likelihood of deterioration with more negative SE (Fig. 3): MD progression (OR = 0.95, 95% CI = 0.93–0.97), TD pointwise progression (OR = 0.96, 95% CI = 0.94–0.97), and MD fast progression (OR = 0.94, 95% CI = 0.91–0.98, *P* < 0.001 for all). Because both the SE and MD slope are more severe as their values decrease (become more negative), an OR <1 reflects a lower SE being associated with a higher likelihood of a more negative MD slope. When we examine the sphere to predict VF progression outcomes with MD adjusted, more negative sphere was significantly correlated with MD progression (OR = 0.95, 95% CI = 0.93–0.96), TD pointwise progression (OR = 0.95, 95% CI = 0.93–0.96), and MD fast progression (OR = 0.94, 95% CI = 0.91–0.97, *P* ≤ 0.001). Pointwise TD slopes were also predicted (Supplementary Fig. S1).

**Figure 4.**
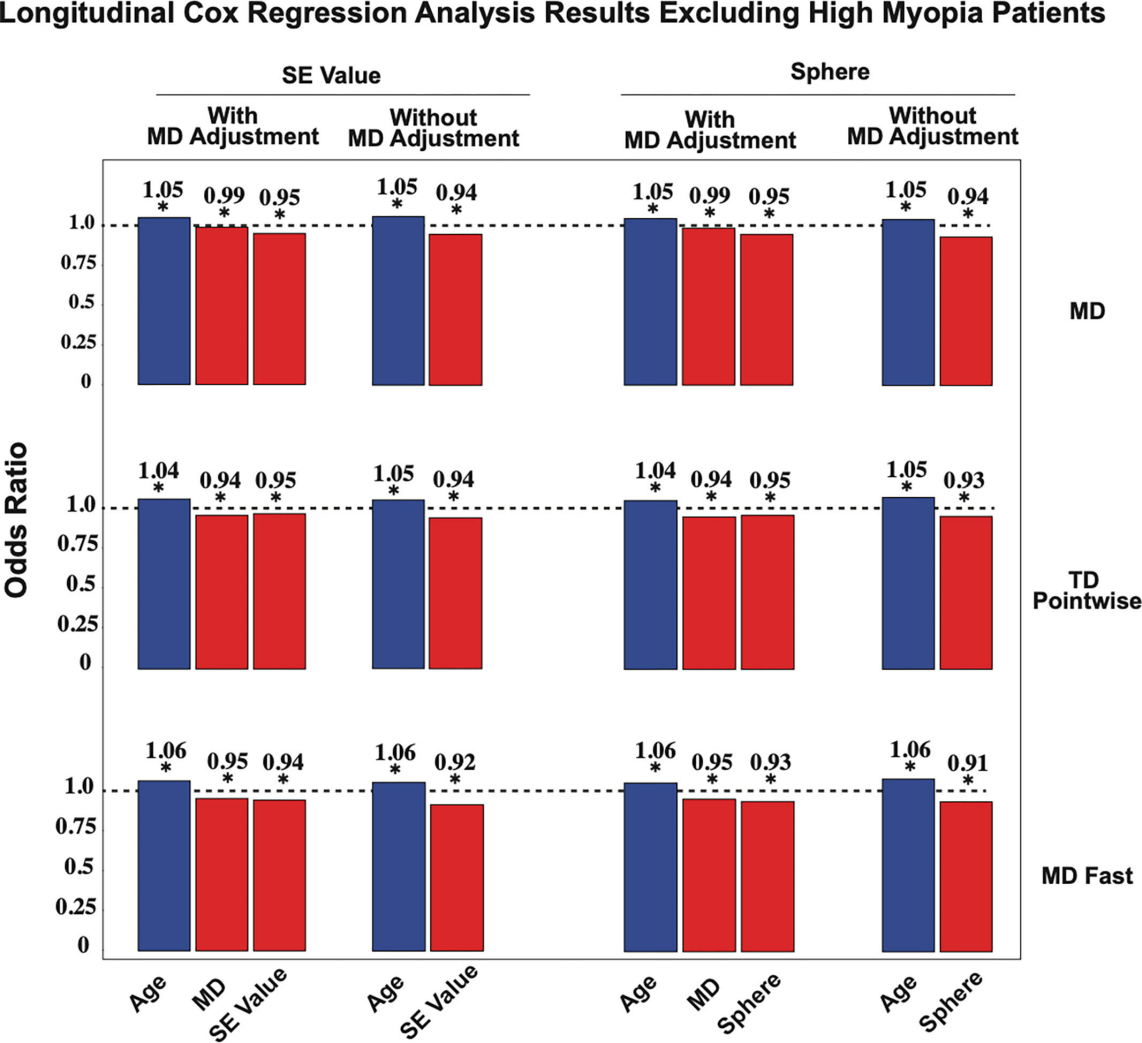
Longitudinal analysis results excluding patients with high myopia wherein regression analysis with both adjustment and no adjustment for MD shown. Statistically significant predictors are denoted by * (*P* < 0.05).

**Figure 5.**
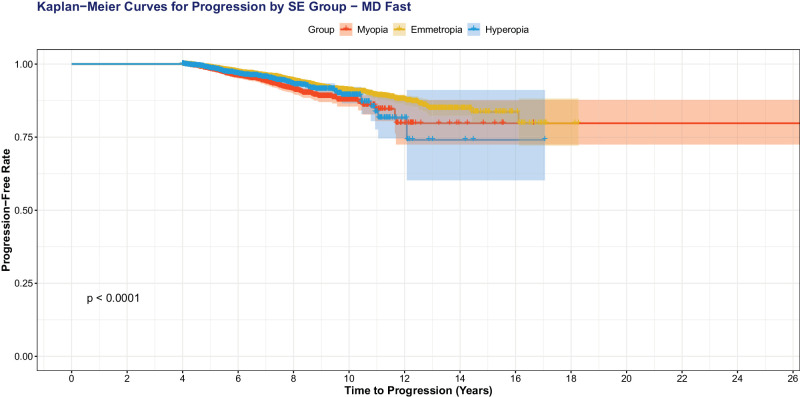
Kaplan-Meier survival curve showing time to progression, as defined by MD fast progression. Myopes progress faster than emmetropes and hyperopes (*P* < 0.001).

**Figure 6.**
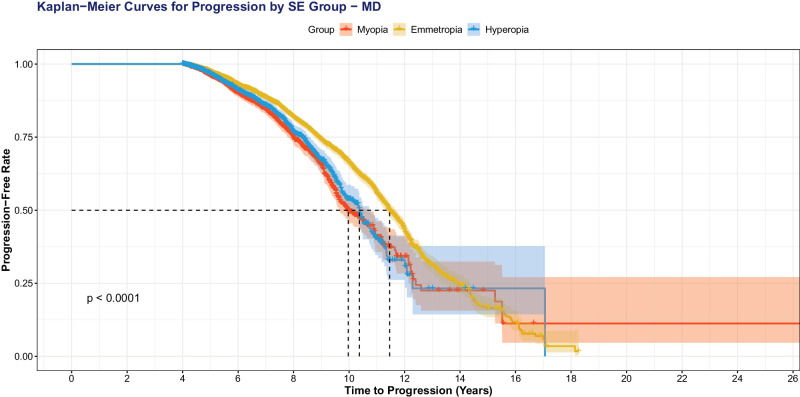
Kaplan-Meier survival curve showing time to progression, as defined by MD progression. Patients with myopia progress faster than emmetropes and hyperopes (*P* < 0.001).

**Figure 7.**
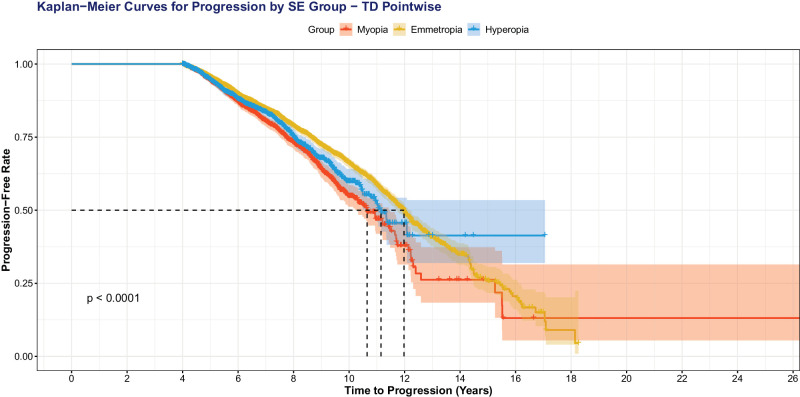
Kaplan-Meier survival curve showing time to progression, as defined by TD pointwise progression. Patients with myopia progress faster than emmetropes and hyperopes (*P* < 0.001).

When we excluded eyes with high myopia (SE ≤−6.00 D; Fig. 4), we still observed that more negative SE was significantly associated with VF progression: MD progression (OR = 0.95, 95% CI = 0.92–0.97, *P* < 0.001), VFI progression (OR = 0.95, 95% CI = 0.93−0.97, *P* < 0.001), and MD fast progression (OR = 0.94, 95% CI = 0.91−0.97, *P* = 0.01). When we used sphere to predict VF progression outcomes with MD adjusted, more negative sphere was significantly correlated with MD progression (OR = 0.95, 95% CI = 0.93−0.96, *P* < 0.001), TD pointwise progression (OR = 0.95, 95% CI = 0.93−0.97, *P* < 0.001), and MD fast progression (OR = 0.93, 95% CI = 0.89−0.97, *P* = 0.01). Kaplan-Meier survival curves with patients separated in myopes (any degree), hyperopes (any degree), and emmetropes demonstrate faster time to progression for all three metrics (*P* < 0.001): MD fast progression (Fig. 5), MD progression (Fig. 6), and TD pointwise progression (Fig. 7).

Similar observations of the association between myopia (more negative SE and sphere) and VF progression were seen without MD adjustment (see Figs. 3, 4).

## Discussion

In this large, multicenter analysis, we used linear regressions in our cross-sectional analysis to characterize the relationship between myopia and regional VF loss in glaucoma suspect eyes. We then used Cox regression for longitudinal survival analyses to characterize the relationship between SE and four measures of VF progression: MD progression, TD progression, and MD fast progression. We then repeated our longitudinal analysis (again, using Cox regression) excluding patients with high myopia to investigate the extent to which lower SE is associated with VF progression in patients without extreme myopia.

Our work has two key contributions. First, we demonstrate that more negative refractive error is not only associated with the presence of progression as measured by MD progression and TD pointwise progression but also with fast progression, as measured by MD fast progression. The association of myopia with fast progression has not been reported in prior studies.^19^^,^^20^^,^^30^ Second and more importantly, we have shown that even when only including mild and moderate myopia, more negative refractive error was significantly associated with both normal progression and fast progression. Although prior work has demonstrated a relationship between high myopia and glaucoma progression, the relationship between mild to moderate myopia and glaucoma progression has not been reported in the literature.^12^^,^^14^^,^^17^

The results of our cross-sectional analysis corroborate prior work in finding that increasing myopia is associated with increased VF loss in the paracentral region, shown by our linear regression model adjusted for age and MD. This functional finding is consistent with prior structural work, which has found that in myopic eyes, the RNFL bundle is closer to the fovea than in myopic eyes, and the RNFL bundle is where structural damage predominantly occurs.^11^^,^^31^

High myopia is considered to have a more aggressive disease course than low to moderate myopia, causing a marked change in the fundus even after adolescence, whereas low to moderate myopia may, although not necessarily, stabilize.^32^^–^^34^ Although the relationship between myopia and VF loss or VF progression in myopia is debatable, the association between high myopia and the prevalence of glaucoma is well-established, owing, among other factors, to tilting and deformation of the optic disc and axial length-related stretching and weakening of the lamina cribrosa that may increase the susceptibility of the optic disc to damage from IOP elevation.^35^^,^^36^ Because of these structural changes, glaucoma can be difficult to diagnose and can therefore be diagnosed later in patients with high myopia. It is possible that some patients in our cohort demonstrated progression during the study period due to their glaucoma being identified at later stages when the disease is more severe. However, our results suggest that even in low to moderate myopia wherein obvious optic disc or other structural deformity may not be present, myopia may pose an increased risk of VF progression and fast progression in glaucoma, although one cannot rule out a purely optical effect of stimulus minification due to correction of myopia in the spectacle plane.^37^^,^^38^

Fast progression can severely impact visual function in glaucoma.^19^^,^^20^^,^^30^ Numerous studies have attempted to find risk factors for fast progression, but none have reported an association of myopia with fast progression. Benefitted by our large sample size, we are the first to demonstrate that fast progression is associated with myopia, even when we excluded patients with high myopia. Considering the increasing prevalence of myopia, our findings underscore the critical importance of implementing preventive measures to control its progression in young individuals.^2^^,^^3^

Limitations apply to this study. Our SE and sphere values are based on correcting lenses that may not represent the true refractive error for every patient, as those estimates are often made by technicians based on the patient's current pair of glasses. Moreover, technicians may also opt to not correct for cylinder. Moreover, the Glaucoma Research Network does not provide data on whether the patients’ eye sights were CL- or spectacle-corrected; this may be an avenue for future study.

In the absence of diagnostic information in our dataset, patients with lens-related conditions, such as pseudophakia due to cataract surgery, that confound the investigation of the impacts of myopia on posterior segment diseases could not be excluded. Cataracts can induce myopic shift, and SE from pseudophakic eyes may reflect an artificial refraction target based on the intraocular lens, rather than the patient's true biological refractive state.^11^ We are not able to account for these factors as the Glaucoma Research Network does not provide information about pseudophakia; however, limitation in ability to account for pseudophakia is not unique to our study.^39^ Additionally, without diagnostic or IOP information, our ability to conclude that VF progression is due to glaucoma is somewhat limited. However, prior work using this large dataset has also been predicated on the fact that VF loss in cases from the Glaucoma Research Network are likely mostly occurring due to glaucoma, given the dataset's origins.^11^ Although high myopia can cause glaucoma-like VF defects, the fact that our results hold even when excluding patients with high myopia supports our assumptions.^40^

This study showed moderate effect sizes and did not account for differences that may exist in clinical subtypes of glaucoma, such as normal tension glaucoma, congenital glaucoma, primary open-angle glaucoma, etc., nor did it account for structural/physiological factors like cup-to-disc ratio, axial length which structurally quantifies myopic severity, or IOP. Moreover, limited by the Glaucoma Research Network dataset, we did not have the patients’ gender, race, and ethnicity information, which are also known to be correlated with myopia, and were therefore unable to account for these factors in our analysis.^41^ Future work may also consider whether concurrent myopia progression is a risk factor for glaucomatous progression. However, glaucoma specialists assess individual patient risk by using clinical judgment to integrate a variety of factors (e.g. IOP, pachymetry, family history, and glaucoma subtype); our findings suggest that myopia ought to be among the clinician's many considerations.^42^ This is particularly true because we demonstrate an association between myopia and fast progression. Due to data availability, our ability to draw definitive conclusions about the underlying causes of VF progression in our sample is limited. However, our work establishes a clear association between myopia and VF progression which may be an important pattern to identify in clinical settings for better prognostication, monitoring, and development of treatment plans for glaucoma in relevant patients. As integrated risk models for glaucoma continue to be developed, our work demonstrates that myopia may be a valuable predictive factor to include.^43^^,^^44^

This analysis used a large multicenter cohort to explore the relationship between myopia and regional VF loss and progression in glaucoma suspect eyes. Consistent with prior work, we find that myopia is associated with VF loss in the paracentral region of the VF. We also find that myopia is not only associated with a higher risk of the presence of VF progression but also with fast progression, which can be disastrous to the patients’ quality of life. More importantly, when excluding high myopia and only examining mild and moderate myopia, a more negative refractive error is still predictive of both normal progression and fast progression. Our findings regarding the association of mild and moderate myopia with normal and fast progression likely due to glaucoma highlight the importance of controlling myopia in young patients, whose degree of myopia may worsen with age, and potentially heightening clinical surveillance of patients with glaucoma and only mild degrees of myopia.

## Supplementary Material

Supplement 1
