# Supplementary figures and images for: The Impact of Myopia on Regional Visual Field Loss and Progression in Glaucoma
Source: Transl Vis Sci Technol. 2025 Sep 24;14(9):34. doi: 10.1167/tvst.14.9.34 (PMC12476162; doi:10.1167/tvst.14.9.34)

**Supplemental Figure 1.** Age-adjusted pointwise TD slope predictions based on baseline SE.

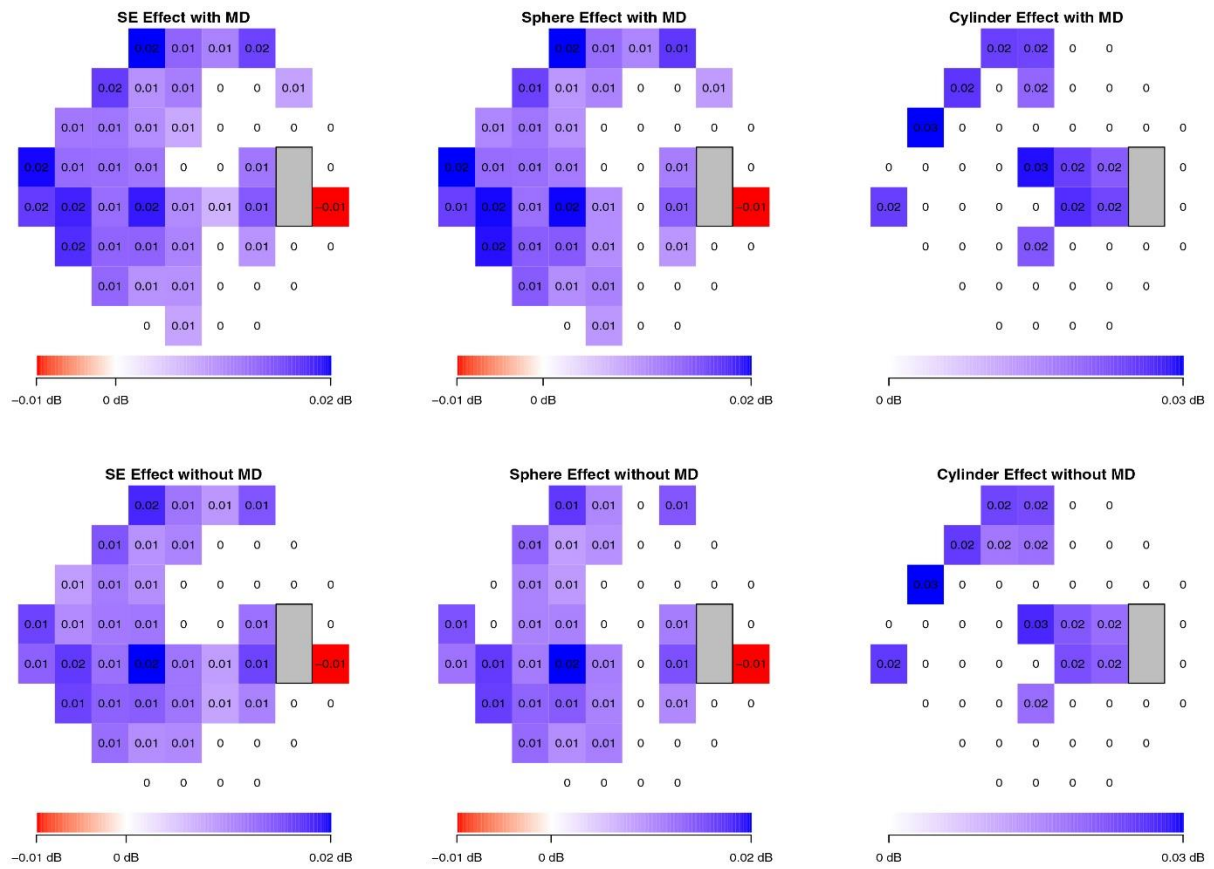

Supplement: Supplement 1 [file tvst-14-9-34_s001.pdf]
